# Type 2 Diabetes Mellitus Intersects With Pancreatic Cancer Diagnosis and Development

**DOI:** 10.3389/fonc.2021.730038

**Published:** 2021-08-16

**Authors:** Xiaoye Duan, Weihao Wang, Qi Pan, Lixin Guo

**Affiliations:** ^1^Department of Endocrinology, Beijing Hospital, National Center of Gerontology, Institute of Geriatric Medicine, Chinese Academy of Medical Sciences, Beijing, China; ^2^Graduate School of Peking Union Medical College, Chinese Academy of Medical Sciences, Beijing, China

**Keywords:** type 2 diabetes mellitus, pancreatic cancer, hyperglycemia, insulin resistance, screening strategy, hypoglycemic therapy

## Abstract

The relationship between type 2 diabetes mellitus (T2DM) and pancreatic cancer (PC) is complex. Diabetes is a known risk factor for PC, and new-onset diabetes (NOD) could be an early manifestation of PC that may be facilitate the early diagnosis of PC. Metformin offers a clear benefit of inhibiting PC, whereas insulin therapy may increase the risk of PC development. No evidence has shown that novel hypoglycemic drugs help or prevent PC. In this review, the effects of T2DM on PC development are summarized, and novel strategies for the prevention and treatment of T2DM and PC are discussed.

## Introduction

In recent years, the incidence of pancreatic cancer (PC) in the world has increased annually. PC has become the third leading cause of cancer-related death in the United States and the fourth in Japan (1). Despite considerable efforts in diagnosis and treatment, the 5-year survival rate has increased to only 10% (1). Because of nonspecific symptoms and a lack of screening recommendations, the vast majority of patients with PC are diagnosed at a late stage, and there is no opportunity for surgical intervention (2). According to data from Chinese Pancreatic Surgery Association, the 5-year overall survival rate of pancreatic cancer was is only 7.2% and the incidence of pancreatic cancer is expected to soar to the second place by 2030 in China (3). Unfortunately, early diagnosis rate of pancreatic cancer is only 5%. The proportion of estimated new cases of pancreatic cancer in China showed obvious regional characteristics, which is consistent with the result that the incidence and mortality increased from low to high urbanization areas in China, and the prevalence of diabetes increased from underdeveloped to developed region (4).

Type 2 diabetes mellitus (T2DM) is considered a risk factor for various malignant tumors, such as hepatocellular cancer, breast cancer, ovarian cancer, endometrial cancer, and gastrointestinal cancer. The incidence of cancer in patients with T2DM has increased by 10%, comparing the public population (5–7). Approximately 50% of patients with PC develop T2DM or impaired glucose tolerance at the very beginning (8). T2DM is a known risk factor for PC, and new-onset diabetes (NOD) may be an early manifestation of PC (9–11). Therefore, T2DM, especially NOD, may be a clue to early detection of PC and may improve the prognosis of this intractable malignant tumor.

However, the incidence of T2DM is too high to justify screening all patients with the condition for PC: the cost-benefit ratio does not justify such widespread use of medical resources. Additional risk stratification is needed in patients with T2DM. In this review, we discuss the mechanism of the relationship between T2DM and PC, update the literature about risk factors and biomarkers of PC in patients with T2DM, and summarize PC prevention and treatment strategies.

## Multiple Underlying Mechanisms Connect T2DM and PC

The mechanisms connecting T2DM with the formation and development of PC are multilayered and complex. Hyperglycemia, hyperinsulinemia, insulin resistance, chronic inflammation, and genetic factors all contribute to the association between these conditions (12).

### Hyperglycemia and PC

**Figure 1:**
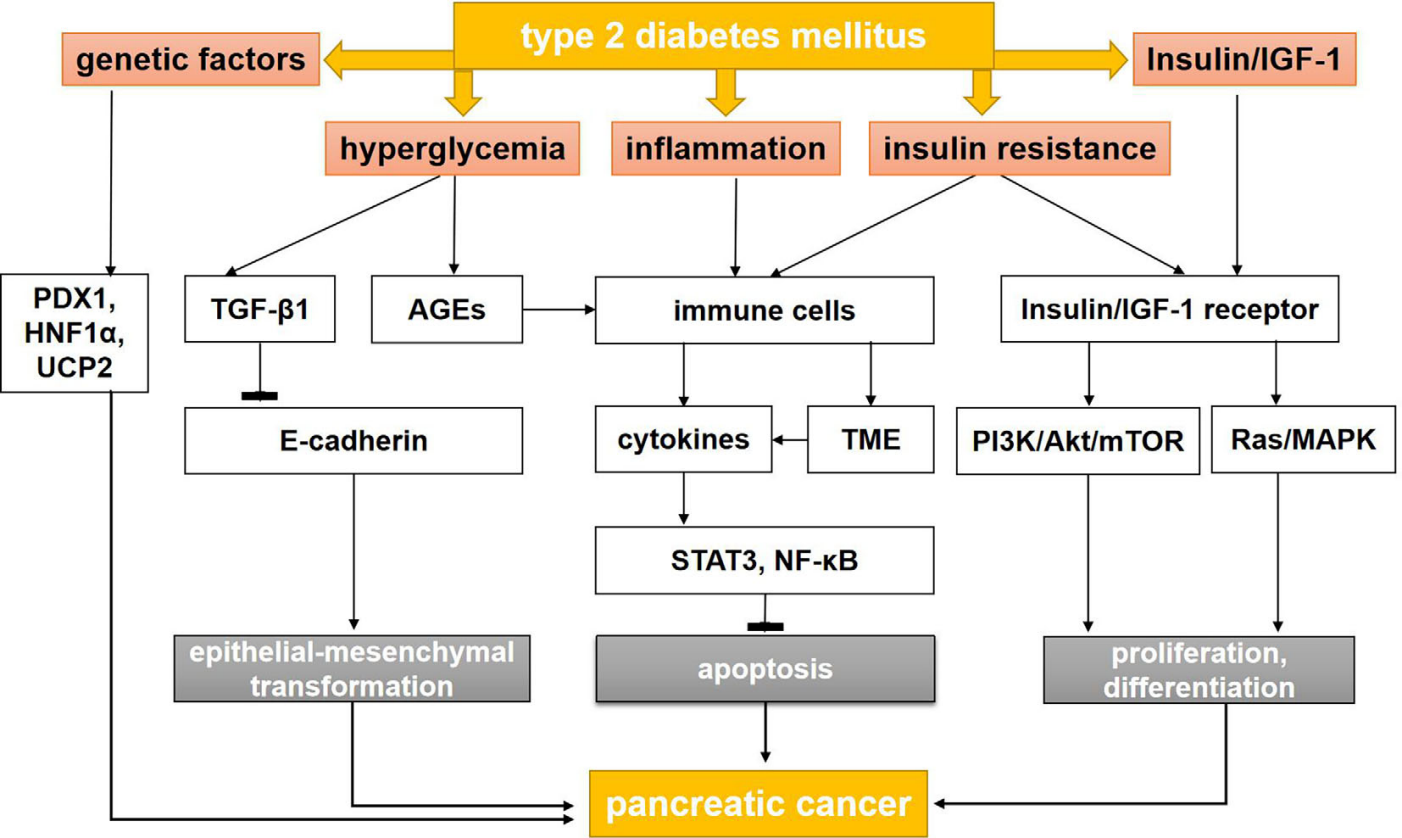
The mechanisms between type 2 diabetes mellitus and pancreatic cancer. AGEs, advanced glycation end products; AMPK, adenosine monophosphate protein-activated kinase; IGF-1, insulin-like growth factor-1; LKB, liver kinase B; MAPK, mitogen-activated protein kinase; mTOR, mammalian target of rapamycin; NF-κB, nuclear factor kappa B; PI3K, phosphatidyl inositol-3 kinase; STAT3, signal transducer and activator of transcription 3; TGF-β1, transforming growth factor-β1; TME, tumor microenvironment; PDX1, pancreatic and duodenal homeobox-1; HNF1A, HNF1 Homeobox A; UCP2, uncoupling protein 2.

In T2DM, hyperglycemia is caused by long-term excessive hepatic gluconeogenesis, decreased insulin activity, low peripheral glucose uptake, and changes in insulin signaling (13, 14). These events can cause cancer, especially PC (5–8) (**Figure 1**). In fact, patients can remain asymptomatic for many years, with undiscovered glucose intolerance and transient hyperglycemia. This time of prediabetes greatly increases the likelihood of developing PC (15, 16). One possible mechanism is the activation of the transforming growth factor-β1 (TGF-β1) pathway by glucose, which results in a decrease in the level of E-cadherin in pancreatic ductal cells and a significant mesenchymal phenotype that promotes tumor growth and metastasis (17). Hyperglycemia may also increase genetic instability and lead to *KRAS* mutations by activating O-GlcN acetylation and nucleotide deficiency (17, 18). Finally, the mTOR pathway controls protein synthesis and autophagy, and its deregulation is associated with diabetes and PC (19–23). Interestingly, inhibition of mTOR can reduce tumorigenesis in *KRAS*-dependent PC.

The tumor-promoting effect of N-carboxymethyllysine was found. N-carboxymethyllysine is a RAGE ligand and a major AGE in pancreatic cancer cell lines. The researchers found that PC was observed in eight (72.7%) of the 11 mice treated with N-carboxymethyllysine but in only one mouse (9.1%) in the control group (25). N-carboxymethyllysine upregulated the expression of RAGE in a concentration- and time-dependent manner, activated leukocyte cell adhesion molecule, and promoted the growth of PC cells (25). Reducing AGEs may be a good way to prevent PC.

Prospective cohort and case-control studies have shown that hyperglycemia is associated with increased free radical formation and may lead to the development of advanced glycosylation end product (AGEs), which may increase inflammation (24). The use of exogenous AGE in PC-susceptible mice can upregulate the expression of the AGE receptor (RAGE) in pancreatic intraepithelial neoplasia and greatly stimulate the development of invasive PC (25).

In 2018, Rahn et al. (17) explored the role of hyperglycemia in the malignant transformation of pancreatic ductal epithelial cell (PDEC), the occurrence and maintenance of cancer stem cells (CSCs), and the promotion of cancer-related epithelial-mesenchymal transformation (EMT). Hyperglycemia did not affect the mesenchymal phenotype of Panc-1 cells but did increase the characteristics of CSCs. In addition, in another study using H6c7-KRAS cells, high glucose stimulated the expression of a TGF-β1 signal and decreased the expression of E-cadherin, increased the expression of nestin, and increased the number of polyclonal cells in a TGF-β1–dependent manner (26). This study also found that decreased E-cadherin was detected in the pancreatic duct of hyperglycemic, but not normoglycemic, mice. These findings suggest that hyperglycemia promotes the acquisition of PDEC mesenchymal and vascular stem cell characteristics by activating TGF-β1 signaling, which may explain how T2DM promotes PC (**Figure 1**).

In addition, hyperglycemia also produce a large number of reactive oxygen species (ROS) (27–29) and reduce the activity of antioxidant enzymes (30, 31) to promote mitosis and stimulate cell proliferation. Luo et al. (28) found that the inactivation of the JNK pathway caused by the increase in ROS levels has a pivotal role on high glucose-induced cell proliferation. Their findings indicated that ROS stimulates proliferation of pancreatic cancer cells under high glucose conditions *via* inactivating the JNK pathway.

### Hyperinsulinemia, Insulin Resistance, and PC

T2DM is characterized by insulin resistance (IR) with hyperinsulinemia and high levels of insulin-like growth factor (IGF)-1 (32–35). In patients with T2DM, IR can lead to hyperinsulinemia through serine phosphorylation of insulin receptor substrate proteins, thus activating protein kinase C and the mTOR complex/S6K and so participating in the downregulation of the insulin signal (36, 37). Insulin can reduce the production of IGF binding proteins 1 and 2 in the liver, both of which have high affinity for IGF-1 and IGF-2, thus increasing the levels of free IGF-1 in circulating blood (36–39).

Most cancer cells highly express insulin and IGF-1, because they are important members of the tyrosine kinase class of membrane receptors and are highly homologous to tyrosine kinase oncogenes (39–44). When insulin and IGF-1 bind to their receptors, they can mediate signal transduction, activate important intracellular signaling pathways—including Ras/Raf/MAPK and PI3K/Akt/mTOR pathways—and lead to the development of PC (45, 46).

Many studies have shown that IGF-1 has a stronger mitotic and anti-apoptotic effect than insulin (47–49). In addition, studies have indicated that cancer cell proliferation increases in a dose-dependent manner with increasing concentrations of IGF-1. Activation of the IGF-1 signal pathway leads to increased PC cell proliferation, invasion, and angiogenesis and to decreased apoptosis (50–53).

### Inflammation and PC

Inflammation may increase the risk of PC in patients with T2DM. In patients with T2DM, insulin resistance and hyperinsulinemia often occur, accompanied by abundant adipocytes and a large amount of inflammatory cell infiltrating the pancreas tissue (54–56). The high glucose and fat diet may accelerate the inflammatory response by increasing oxidative stress and activating transcription factors, such as nuclear factor kappa B (NF-κB) and activator protein 1, which leads to the development of genomic aberration and carcinogenesis (57–59).

Inflammatory cytokines, ROS, and mediators of inflammatory pathways, such as cyclooxygenase-2 and NF-κB, are closely related to the STAT3 pathways. STAT3 and NF-κB signaling pathways are proven inhibitors of apoptosis and promoters of cell cycle progression. They also downregulate the expression of E-cadherin to induce EMT. During the inflammatory response, immune cells may directly promote the growth and progression of PC by releasing a large number of cytokines and growth factors into the microenvironment. The environment around the tumor is called as tumor microenvironment (TME), including Carcinoma-associated fibroblasts, endothelial cells and immune cells, which plays an significant role in growth, invasion and metastasis of pancreatic cancer. Inflammation change the TME and break balance of cancer cells in growth and apoptosis (60–63) (**Figure 1**).

### Genetics Factors Driving DM and PC

A genome-wide association study has identified the relationship between diabetes and PC. Some pancreatic developmental genes, such as *NR5A2*, *PDX1*, and *HNF1A*, have been identified as susceptibility factors for PC in T2DM patients. Heterozygous mutations in some of these genes, such as *PDX1* and *HNF1A*, also lead to different types of monogenic diabetes in young people (types 4 and 5). Some variants in *PDX1* and *HNF1A* are also associated with an increased risk of T2DM (64, 65), obesity, or hyperglycemia (66).

The antioxidant mitochondrial uncoupling protein 2 (UCP2) controls pancreatic development and insulin secretion (67). UCP2 is overexpressed in PC tumors compared with normal adjacent tissues, indicating that its overexpression is a biomarker of poor prognosis. However, other recent studies using the PC cell line MiaPACA2 have shown that UCP2 can inhibit cancer cell proliferation and tumorigenesis (68). This effect is mediated by the retrograde mitochondrial signal on Warburg, which redirects mitochondrial function to oxidative phosphorylation rather than to glycolysis (69). Additional analysis is needed to clarify the differences between these two studies involving UCP2. Taken together, these data suggest a link between genes that control DM and PC.

## Screening Strategies for Early Diagnosis of PC in Patients With T2DM

NOD may be an early sign of PC, and a sudden increase in blood glucose in patients with previously well-controlled T2DM may also be a sign of PC (70). However, universal screening of PC in all elderly patients with NOD is difficult to achieve and not cost effective. In recent years, many studies have proposed different strategies to stratify T2DM groups and facilitate targeted screening (71–74).

A prospective observation cohort study initiated by the Consortium for the Study of Chronic Pancreatitis, Diabetes, and Pancreatic Cancer proposed a new approach (define, enrich, find) to clarify the population at high risk of PC and to detect lesions in the high-risk groups (75). Patients older than age 50 years were divided into high-, medium-, and low-risk groups using the Enriched New-Onset Diabetes Score for Pancreatic Cancer (END-PAC). This scoring model provides a reference for early clinical screening of PC (76). Elderly patients with weight loss [low body mass index (BMI)] and rapidly rising blood glucose levels in a short period may be the target population for early screening of pancreatic cancer (77). Other indicators for screening PC in patients with T2DM include BMI; age of T2DM onset; hepatitis B virus infection; and total bilirubin, alanine aminotransferase, creatinine, apolipoprotein A1, and leukocyte (WBC) levels (78). Fatigue and depression caused by elevated interleukin-6, combined with severe weight loss (>10%) and NOD, may represent paraneoplastic syndrome and be early manifestations of PC (79).

In addition to contributing to risk stratification, the development of biomarkers to distinguish PC-associated DM is expected to be an important aspect of PC screening. Studies have shown that the numerous molecules, described in the following sections, may be effective biomarkers for an early diagnosis of PC (**Table 1**).

**Table 1:** Risk factors, early signs, and biomarkers for pancreatic cancer in patients with type 2 diabetes mellitus.

Risk factor	NOD (≤2-year duration) Elderly onset (≥65 years)
Early sign	Body weight loss Rapid exacerbation of glycemic control
Biomarker	sTNF-αR2 OPG VNN1 IGF Circulating RNA

NOD, new-onset diabetes; sTNF-αR2, soluble receptor 2 of tumor necrosis factor-α; OPG, osteoprotegerin; VNN1, Vanin-1; IGF, Insulin-like growth factor.

### Carbohydrate Antigen 19-9

The level of carbohydrate antigen 19-9 (CA19-9) secreted by cancer cells in patients with NOD may be a reliable indicator for predicting PC. However, CA19-9 has a high false-positive rate; any condition that causes inflammation of the pancreas increases the CA 19-9 level. One study has shown that the CA19-9 level is of little significance in screening PC in NOD, because the positive predictive value and sensitivity are zero, and the false-positive rate is 9% (80). However, the sample size of this study was small, so the conclusions cannot be applied to the entire population. Other studies have shown that, in the first 2 years of NOD, CA19-9 can be used as a cost-effective approach to detect small PC lesions that cannot be detected on imaging (81, 82).

### Soluble Receptor 2 of Tumor Necrosis Factor-o

During the systemic inflammatory response to PC, C-reactive protein (CRP) levels can increase. Tumor necrosis factor-α (TNF-α) is the upstream regulator of CRP. Grote et al. (83) found that an increase in soluble TNF receptor 2 (sTNF-R2), significantly increases the risk of PC in patients with diabetes. In the diabetes arm of the study, the odds ratio of PC when the sTNF-R2 doubled was 4.76 (95% CI, 1.11–20.37); in the arm without diabetes, the odds ratio was only 1.12 (95% CI, 0.73–1.72) (83).

### Osteoprotegerin

Osteoprotegerin (OPG) is a soluble decoy receptor of TNF-related apoptosis-inducing ligand, which belongs to the TNF receptor superfamily. Shi et al. (84) found that serum OPG was significantly increased in patients with PC-related DM. The sensitivity of serum OPG in identifying PC in patients with NOD was 68%; the specificity was 73.9%; and the area under the curve (AUC) was 73.7%.

### Vanin-1

The enzyme vascular non-inflammatory molecule-1 (vanin-1) is highly expressed at gene and protein level in many organs. Recently, many researches have elucidated the role of vanin-1 under physiological conditions in relation to oxidative stress and inflammation, which is important in the pancreatic microenvironment (85). Huang et al. (86) identified vanin-1 (VNN1) as a potential biomarker for PC, using microarray analysis of the peripheral blood in patients with PC-associated DM compared with T2DM (84). Kang et al. (87) also explored the functional mechanism of VNN1 in PC-associated DM and found that overexpression of VNN1 in tumor tissues can decrease glutathione concentration and increase ROS, thus aggravating paraneoplastic islet dysfunction.

### Circulating RNA

Recently, circulating RNAs have become research hotspots as noninvasive biomarkers for the early detection of PC (88). PC cells release a large amount of RNA into the bloodstream. These RNAs can effectively resist the RNA enzyme, thus increasing the expression level in the serum. Dai et al. (89) reported a microRNA panel (miR-483-5p, miR-19a, miR-29a, miR-20a, miR-24, miR-25) that distinguished PC-related DM from T2DM with an AUC of 0.887.

Although a number of studies have reported biomarkers for PC-related DM, most of them are case-control studies with limited sample sizes. Future studies must verify the role of these discussed biomarkers in distinguishing T2DM with PC *versus* without PC in larger samples.

## Effects of Antidiabetic Therapy on PC

Some antidiabetic medications may have an impact on PC development, progression, and outcome because of their direct effects on the key factors mediating the association between T2DM and PC. The safety of antidiabetic medications with regard to PC risk is discussed in the following sections.

### Insulin Therapy

Insulin therapy is usually necessary to treat T2DM in the long term. However, abundant research has shown that insulin therapy may increase the incidence of PC (90, 91).

To explore the risk relationship between insulin therapy and PC, Bosetti et al. (92) analyzed 15 case-control studies, which included 8,305 patient cases and 13,987 controls. Studies indicated that short-term insulin use (<5 years) was independently associated with a higher risk of PC (odds ratio [OR] = 5.6, 95% CI, 3.75–8.35), whereas long-term insulin use (≥15 years) was not (OR = 0.95, 95% CI, 0.53–1.70). At the same time, studies also showed that long-term oral antidiabetic use (≥15 years) in patients with T2DM might reduce the risk of PC (OR = 0.31, 95% CI, 0.14–0.69).

In 2018, Lee et al. (93) conducted a population-based study comparing PC risk in patients expose to antidiabetic drugs *versus* no drug exposure. The study concluded that, among several kinds of antidiabetic drugs, insulin alone was associated with an increased risk of PC (hazard ratio [HR] = 2.86, 95% CI, 1.43–5.74). The conclusion is similar with the research by Liu et al. (94), a case–control study using 12 years of data from Taiwan’s National Health Insurance Research Database. The association between insulin use and high pancreatic cancer risk is significant.

Wang et al. (95) also found that insulin can promote the proliferation and glucose utilization of PC cells by activating ERK and PI3K and by increasing the expression of MMP-2. Insulin promotes migration and invasion in PC by activating the MMP-2 signal pathway. In addition, insulin induces phosphorylation of ERK and PI3K/Akt, which indicates that insulin can stimulate the Ras/Raf/MAPK and PI3K/Akt pathways and accelerate tumorigenesis and development (**Figure 1**). In summary, insulin use is associated with an increased risk of PC, so patients with T2DM who have a high risk of PC may not be candidates for insulin treatment. While insulin treatment was imperative for the patients with insulin secretion absolutely insufficient. For clinical physicians, we should pay attention to the risk of PC during long-term treatment with insulin and screen early PC in islet β-cell dysfunction patients with long-term treatment with insulin. We need more evidence for PC risk for patients with long-term treatment with insulin in the further research.

### Metformin Therapy

Metformin is the cornerstone treatment of diabetes. Retrospective studies have shown that metformin can improve the survival of patients with T2DM and PC. During the past 5 years, numerous studies have suggested that metformin can reduce the risk of PC (96–99).

In the analysis of case-control studies by Bosetti et al. (100), long-term oral metformin use (≥15 years) reduced the risk of PC in patients with T2DM (OR = 0.31, 95% CI, 0.14–0.69). In 2018, Lee et al. (101) conducted a population-based study to assess the effects of T2DM and antidiabetic drugs on PC risk. That study identified metformin, among the antidiabetic drugs studied, as an independent risk factor for PC (HR = 0.86, 95% CI, 0.77–0.96). However, patients who received metformin combined with a thiazolidinedione or with dipeptidyl peptidase-4 inhibitors had lower risks of PC than patients receiving metformin alone. There are several clinical trials about metformin on PC treatment, such as metformin combined With Chemotherapy. Although addition of metformin does not improve outcome in patients with advanced PC treated with gemcitabine and erlotinib, future research should include studies of more potent biguanides, and should focus on patients with tumors showing markers of sensitivity to energetic stress, such as a lack of function of AMP kinase (102).

Currently, most scholars believe that metformin can reduce the risk of PC, because metformin can activate the liver kinase B1 (LKB1)–adenosine monophosphate protein-activated kinase (AMPK) pathway, which can not only promote cell energy production and inhibit liver glucose production but also inhibit the signal pathway of cancer cell proliferation (103). As a tumor suppressor, LKB1 can activate AMPK, which is a potent inhibitor of mTOR complex 1, and disrupt cross-talk between insulin/IGF-1 receptor and G protein–coupled receptors, thus regulating protein synthesis and replication. More importantly, metformin may play a role in the development of PC stem cells through the mTOR pathway (23, 104–107).

In a study evaluating the effect of metformin on PC, cancer stem cells (Alk4, nodal, activin, and Smad2) and pluripotency-related RNA proteins (Nanog, Oct4, and Sox2) changed significantly after metformin treatment. These changes may be due to the inhibition of nicotinamide-adenine dinucleotide dehydrogenase and the production of free ROS, which would directly increase the damage to PC stem cells (108). Ma M et al. found that metformin significantly inhibited proliferation and viability, induced apoptosis of pancreatic cancer cells, which was more pronounced in low-glucose than in high-glucose group, and metformin may play protective effect by suppressing glycolysis and inducing energy stress *via* up-regulation of miR-210-5p (109).

These studies have shown that metformin can reduce the risk of PC and activate the LKB1/AMPK pathway, thus inhibiting cell proliferation by mTOR. Therefore, metformin is expected to become part of the standard treatment for patients with PC.

### Incretin-Based Medicines

The use of incretin-based medicines—glucagon-like peptide 1 receptor agonists (GLP1-RAs) and dipeptidyl peptidase-4 inhibitors (DPP4is)—is increasingly popular. Some studies in animal models have been speculated that the chronic overstimulation of GLP1 receptors in exocrine pancreatic cells may induce pancreatitis, ultimately increasing the risk of PC (110, 111). However, this hypothesis has not been supported by evidence from clinical trials (112, 113).

Monami et al. (112) analyzed 113 trials, and 15 studies that reported at least one event. Those 15 studies enrolled 14,866 and 12,849 patients in GLP1-RA and comparator groups, respectively, and the number of reported PCs was 24 for GLP1-RAs and was 23 for comparators (Mantel-Haenszel odds ratio [MH-OR] for PC with GLP1-RA treatment = 0.94, 95% CI, 0.52–1.70, *P* = 0.84). Similar results were obtained in a post-hoc analysis excluding comparisons with DPP4is (MH-OR = 0.93, 95% CI, 0.51–1.69, *P* = 0.80).

In 2020, Nreu et al. (113) analyzed 43 randomized, controlled trials that met the following inclusion criteria: at least 52 weeks in duration, and comparison of a GLP1-RA *versus* any non–GLP1-RA treatment in patients with T2DM and PC. They found that GLP1-RA use showed no association with PC (MH-OR = 1.28, 95% CI, 0.87–1.89, *P* = 0.20) (107).

Currently, no clear evidence of risk for PC has been observed with the use of incretin-based medications. Data about the relationship between incretin-based medicines and PC may be too scarce to draw any conclusion.

### Sodium-Glucose Cotransporter 2 Inhibitors

Sodium-glucose cotransporter 2 (SGLT2) inhibitors represent a novel class of oral antidiabetic drugs that help maintain glycemic control by decreasing the reabsorption of glucose and increasing the excretion of urinary glucose (114). In addition to substantial cardiovascular benefits, anti-tumor benefits or the safety of SGLT2 inhibitors have been considered by the public. Scafoglio et al. (115) found that SGLT2 was functionally expressed in pancreatic carcinomas and that SGLT2 inhibitors blocked glucose uptake and reduced tumor growth and survival in a xenograft model of PC. These findings suggest that SGLT2 inhibitors may be useful for cancer therapy.

In 2019, Tang et al. (116) undertook a study to systematically evaluate the association between SGLT2 inhibitors and pancreatic safety in patients with T2DM. Of the 35 trials, involving 44,912 patients with T2DM, 40 PC events (in 18 trials and 27,806 patients) were reported during a median follow-up of 52 weeks. SGLT2 inhibitors were not associated with PC (OR=1.34; 95% CI, 0.71–2.54; very-low-quality evidence) (116).

## Conclusion

PC is highly aggressive and lethal malignancy, and T2DM is the most common metabolic disease. T2DM is a risk factor for PC. Conversely, NOD may be a sign and consequence of PC. Screening in patients with NOD combined with assessment of risk factors and biomarkers may be an important way to improve the early diagnosis of PC. The mechanisms that contribute to the relationship between PC and diabetes include insulin resistance, hyperinsulinemia, hyperglycemia, and chronic inflammation. Metformin, insulin, GLP1-RAs, DPP4is, and SGLT2 inhibitors are common drugs that treat T2DM. Studies have shown that metformin can reduce the risk of PC, whereas insulin therapy is associated with a higher risk of PC. Therefore, metformin may be used to prevent the development of malignant lesions and is expected to become an anticancer agent. T2DM-related studies will likely be crucial to improve the morbidity and mortality associated with PC.

## Author Contributions

XD consulted literatures and wrote the manuscript. LG designed the review. WW and QP assisted with writing and revised the manuscript. All authors contributed to the article and approved the submitted version.

## Funding

This work was supported by the National Natural Science Foundation of China (grants 81670763 and 81471050).

## Conflict of Interest

The authors declare that the research was conducted in the absence of any commercial or financial relationships that could be construed as a potential conflict of interest.

The handling editor and the reviewer (YL) declared a shared affiliation with the authors at time of review.

## Publisher’s Note

All claims expressed in this article are solely those of the authors and do not necessarily represent those of their affiliated organizations, or those of the publisher, the editors and the reviewers. Any product that may be evaluated in this article, or claim that may be made by its manufacturer, is not guaranteed or endorsed by the publisher.
